# Therapeutic potential of *Astragalus*-based Eefooton in patients with chronic kidney disease: from clinical to bench study

**DOI:** 10.7150/ijms.102280

**Published:** 2025-01-01

**Authors:** Kuo-Cheng Lu, Ko-Lin Kuo, San-Chiang Wu, Chih-Hui Lin, Cheng-Ju Lin, Yi-Chou Hou, Jin-Shuen Chen

**Affiliations:** 1 Division of Nephrology, Department of Medicine, Taipei Tzu Chi Hospital, Buddhist Tzu Chi Medical Foundation, New Taipei City 231, Taiwan.; 2 Division of Nephrology, Department of Medicine, Fu Jen Catholic University Hospital, School of Medicine, Fu Jen Catholic University, New Taipei City 243, Taiwan.; 3 Wu San-Chiang Medical Clinic, No. 240, Xianzheng Rd., Lingya Dist., Kaohsiung City, Taiwan.; 4 Gerent Biotech R&D center, 2 F., No. 173, Sec. 4, New Taipei Blvd., Xinzhuang Dist., New Taipei City 242032, Taiwan.; 5 Division of Nephrology, Department of Internal Medicine, Cardinal Tien Hospital, School of Medicine, Fu Jen Catholic University, New Taipei City 242, Taiwan.; 6 Kaohsiung Veterans General Hospital Director, Department of Medical Education and Research Chairman of International Affairs Committee, Taiwan Society of Nephrology, Kaohsiung City 813414, Taiwan.

**Keywords:** Chronic kidney disease (CKD), Eefooton (EFT), estimated Glomerular Filtration Rate (eGFR), Fibrosis, poly-ADP-ribose polymerase-1 (PARP-1), Traditional Chinese Medicine (TCM)

## Abstract

**Objective:** Chronic kidney disease (CKD) is a global health concern, and recent clinical evidence suggests the potential of traditional Chinese medicine (TCM) to slow CKD progression. This offers alternative strategies for CKD patients, mitigating risks related to polypharmacy and adverse drug reactions. Our self-controlled, prospective study aims to assess the impact of Eefooton (EFT), a TCM-based regimen, on kidney health in stage 3-5 CKD patients. Additionally, we conduct a cell culture study to explore the potential mechanisms of EFT in protecting renal function.

**Materials and methods:** Between 2021 and 2022, 75 stage 3-5 CKD patients (56% males; mean age 68.20y) at Kaohsiung Veterans General Hospital and Wu San-Chiang Medical Clinic received six months of EFT treatment alongside conventional CKD medications. The primary outcome assessed was the change in estimated glomerular filtration rate (eGFR) at 6 months, with secondary outcomes including kidney size and blood biomarker changes. Adverse events were monitored. In an *in vitro* study, EFT effects on HK-2 cell viability and clonogenicity, as well as analysis of apoptosis and fibrosis-related proteins through Western blot, were investigated.

**Results:** Median eGFR significantly improved from 34.37 ± 13.58 to 42.47 ± 18.82 mL/min/1.73 m^2^ (p < 0.001) at month 6 post-treatment. Notably, improvements were observed across different baseline CKD stages (stage 3: p < 0.001, stage 4: p = 0.037). Ultrasonography scans indicated a slight increase in mean kidney size. *In vitro*, EFT enhanced HK-2 cell viability and increased clonogenicity. Indoxyl sulfate exposure raised cleaved and total PARP-1 activity. Co-treatment with EFT and IS reduced cleaved PARP-1 activity. EFT decreased IS-induced expression of fibrosis-related proteins (α-smooth muscle actin) without affecting apoptosis-related proteins (Caspase 3).

**Conclusions:** When combined with conventional CKD medications, EFT has shown effectiveness in enhancing kidney function in individuals with stage 3-5 CKD, with no reported safety concerns. The PARP-1 inhibition and anti-fibrosis properties of EFT present potential benefits in the context of CKD.

## Introduction

CKD globally leads to significant morbidity and heightened cardiovascular and overall mortality 1. Managing renal dysfunction through pharmacotherapy is intricate. In recent years, increasing evidence suggests the potential of traditional Chinese medicine (TCM) derived from plants as an adjunctive therapy for CKD. This is especially evident in its ability to alleviate the side effects of Western medications and protect kidney function 2-5. The utility of plants being employed for therapeutics relies on the fact that they produce a vast and diverse assortment of organic compounds 6. These compounds and their metabolites may act separately, additively, or in cooperative energy to enhance well-being 7. Lin *et al.*'s study can serve as a roadmap for future research investigating the application of Chinese herbal medicine and uncovering the active components for treating patients with CKD 8.

Eefooton (EFT), a liquid herbal extract formula consisting of *Astragalus membranaceus* (3 g), *Codonopsis pilosula* (3 g), *Ligustrum lucidum* (3 g), *Panax quinquefolius* (1.3 g), and *Rhodiola sacra* (1.3 g), has been identified as an adjuvant therapy for patients with CKD. A previous study demonstrated its ability to decelerate the decline in renal function 5**.** The primary component of EFT, Astragalus propinquus, has been documented to decrease proteinuria while increasing serum albumin and hemoglobin levels in CKD 9. *Astragali Radix* (ARE) has been reported to alleviate proteinuria by suppressing the overexpression of eNOS and inhibiting oxidative injury in rats with adriamycin nephropathy 10. *Astragalus membranaceus* (AM) and its active components successfully lowered fasting blood glucose and proteinuria levels in rats with diabetic kidney disease (DKD). Additionally, they reversed glomerular hyperfiltration and mitigated pathological changes in an early DKD rat model 11-13. *Codonopsis pilosula* (C. pilosula) stems and leaves contain active metabolites such as saponins, flavonoids, terpenoids, and polysaccharides. Aerial parts of *C. pilosula* exhibit stronger antioxidant activity compared to the roots 14. The renoprotective effect is likely mediated by the inhibition of the proinflammatory cytokine TNF-α release 15. Fructus Ligustri Lucidi (FLL), derived from *Ligustrum lucidum* Ait., is a traditional Chinese medicine known for its antioxidant properties 16. Used for decades to strengthen the kidney and liver, pretreatment of UUO mice with hetero-polysaccharide from FLL effectively alleviated glomerulosclerosis and tubulointerstitial fibrosis, indicating its potential to protect kidneys from fibrosis 17. *Panax quinquefolius*, or American ginseng, is a North American native ginseng species known for its potential to treat renal impairment 18. Its key physiologically active components 19 offer advantages, including the enhancement of glomerular endothelial barrier function, reduction of renal cell apoptosis in acute kidney injury (AKI), prevention of excessive extracellular matrix (ECM) buildup in renal tubular cells, inhibition of apoptosis in glomerular mesangial cells, and mitigation of podocyte damage 20. Ginsenoside Rb1 (GS-Rb1), an established antioxidant found in the herbal medicine ginseng, is believed to have a protective role in CKD, potentially due to its involvement in mitigating reactive oxygen species (ROS) 21. *Rhodiola rosea*, a perennial plant of the Crassulaceae family, is known for containing over 140 chemical substances, including phenols, flavonoids, and salidroside, mainly found in its roots 22, 23. A study suggests that salidroside exhibits nephroprotective effects in diabetic kidney disease patients by inhibiting apoptosis in proximal renal tubular cells 24.

In this study, our goal was to assess the therapeutic potential of EFT, a Traditional Chinese Medicine (TCM)-based regimen, in patients with stage 3-5 CKD, with a specific focus on its impact on renal function progression. Additionally, we conducted a preliminary cell line study to confirm the potential beneficial effects of the herbal compound EFT on renal tubular epithelial cells.

## Methods

### Study design and participants

This prospective cohort study included participants aged 20-80 with stage 3-5 CKD, characterized by an eGFR < 60 mL/min/1.73 m^2^, who were not undergoing dialysis. The study was conducted at Kaohsiung Veterans General Hospital and Wu San-Chiang Medical Clinic from July 1, 2021, to December 31, 2022. Written informed consent was obtained from all participants, and the study protocol received approval from the Human Ethics Committee of Kaohsiung Veterans General Hospital Institutional Review Board (approval number: KSVGH21-CT5-19). The study was also registered on ClinicalTrials.gov with the identifier NCT04940117.

### Preparation of drugs

Eefooton is a liquid formula of herbal extracts consisting of* Astragalus membranaceus* 3g, *Codonopsis pilosula* 3g, *Ligustrum lucidum* 3g, *Panax quinquefolius* 1.3 g, and *Rhodiola sacra* 1.3g in 20mL water, and has the ISO22000 and hazard analysis and critical control points certifications approved by United Kingdom Accreditation Service. The Certificate number: 2619168001. All plant names have been cross-checked with the MPNS database (http://mpns.kew.org) and compared with the official names in the Chinese Pharmacopoeia. After weighing, the herbs are cleaned thoroughly and placed in storage containers. Then, a tenfold amount of purified water is added. The extraction method is documented in the International Trust Machines file (Name: Eefooton) confidential content.docx.

High-performance liquid chromatography (HPLC) interpretation data employs Astragaloside IV as the quality control parameter, primarily due to its current convenience of access. Reagents, including methanol (99.9%) and acetonitrile (99.9%), are purchased from Merck; ammonia solution (25%) is procured from Youhe Trading Co., Ltd. The standard reference substance, Astragaloside IV, is obtained from the Bureau of Traditional Chinese Medicine, with a purity of over 98%.

### Intervention: Eefooton (EFT)

After being dissolved in 20 mL of distilled water, each dose is designed to support kidney health. In 2010, EFT obtained approval in Taiwan as an adjunct therapy to postpone the onset of dialysis in individuals with progressive CKD 5. During the study, participants, in addition to their standard CKD medications, were instructed to orally consume 20 mL of EFT three times daily for 6 months, following meals. If other medications had been taken previously, a half-hour interval was recommended.

### Quantitative analysis of Astragaloside IV in EFT using HPLC

HPLC-UV analysis was conducted using the Jasco HPLC-PU4180 system, equipped with an AS-4150 autosampler and an MD-4010 UV detector (wavelength range 190-900 nm), along with a column temperature control compartment. The Kromasil 100-5-C18 column (4.6 × 250 mm) was utilized, which is suitable for separating medium to highly polar compounds. The chemicals and solvents used in the following experiments were all HPLC grade.

To prepare the standard solution, 5 mg of Astragaloside IV standard was dissolved in methanol, resulting in a stock solution concentration of 1 mg/mL. The stock solution was then serially diluted with methanol to create standard concentrations of 200, 100, 50, 25, and 10 μg/mL. Each diluted standard solution was filtered through a 0.22 μm membrane filter to ensure purity before injection into the HPLC.

For sample extraction, 0.1 mL of sample was mixed with 1 mL of methanol. The mixture was vortexed for 5 min to enhance dissolution and then sonicated for 10 min to increase extraction efficiency. After sonication, samples were centrifuged at 1,000 g for 10 min at room temperature. Finally, the supernatant was filtered through a 0.22 μm filter to ensure purity for HPLC analysis. The samples were repeated three times.

The HPLC analysis was conducted using a Kromasil 100-5-C18 column (4.6 × 250 mm). The mobile phase consisted of methanol and DI water in an 80:20 ratio. Key operational parameters were as follows: (1) Injection Volume: 20 μL; (2) Flow Rate: 0.8 mL/min; (3) Detection Wavelength: 205 nm; (4) Column Temperature: 40°C.

### Clinical study outcomes

The main focus of the assessment was the alteration in eGFR, recognized as a crucial indicator in clinical practice for evaluating kidney function 25, 26. Secondary outcomes encompassed: 1) variations in kidney size, evaluated through ultrasonography by proficient radiologists; 2) fluctuations in blood biomarkers, such as serum levels of potassium, sodium, calcium, phosphorus, glycated hemoglobin (HbA1c), hemoglobin, and liver enzymes (GOT and GPT); and 3) monitoring of adverse events.

### Renal tubular epithelial cell culture

The HK-2 cell line (ATCC CRL-2190, BCRC 60097, Taiwan) was grown in Dulbecco's Modified Eagle's Medium (DMEM; Gibco, NY, USA) with 10% fetal bovine serum (FBS; Gibco, NY, USA) and 1% penicillin-streptomycin (Gibco, NY, USA) in a 5% carbon dioxide incubator at 37°C. Before use, all cell lines in our study were tested to ensure the absence of mycoplasma contamination.

### HK-2 cells viability (proliferation) assay

Cell viability was evaluated using the CellTiterGlo Luminescent Cell Viability Assay 27. In triplicate, 1,000 cells were seeded in 96-well plates. At specified intervals, viability was determined using the CellTiterGlo assay (Promega, Madison, WI, USA), and bioluminescence was measured with a TECAN Infinite F200 plate reader (TECAN, Maennedorf, Zürich, Switzerland).

To assess the impact of indoxyl sulfate (IS) on the HK-2 cell line, IS was introduced after cells were incubated for 24 hours at concentrations of 0, 250, 500, and 1000 μM for 24 and 48 hours, respectively. Similarly, to evaluate the effect of EFT on HK-2 cell viability, EFT was added at concentrations of 0, 0.116, 0.14, 0.29, 0.58, and 5.8 mg/ml for 24 and 48 hours after seeding.

### Clonogenic assay of HK-2 cells

To evaluate the clonogenicity of EFT on HK-2 cells, visible colonies were observed under a 10X magnification microscope. HK-2 cells were cultured in 6 cm dishes with DMEM (containing 10% FBS) and 1% penicillin-streptomycin at a density of 1 x 10^3^ cells or fewer 28. After 24 hours of incubation, EFT was introduced at concentrations of 0, 0.29, 0.58, 2.9, 5.8, and 58 mg/ml. The cells were then incubated for 1-2 weeks until clusters became visible.

### Western blot analysis for proteins associated with apoptosis and fibrosis

Cells were seeded at a density of 3 × 10**^5^** cells/well in a 6-well plate. After a 48-hour incubation, cell pellets were lysed in RIPA buffer, centrifuged at 14,000× g, and 40 µg of protein extracts from each sample were subjected to SDS-PAGE. Subsequently, they were transferred onto polyvinylidene difluoride membranes. The membranes were blocked in 3% skimmed milk with TBST buffer for 1 hour at room temperature, followed by two 10-minute washes.

Immunoblotting involved probing the membranes with primary antibodies against caspase 3, poly-ADP-ribose polymerase-1 (PARP-1), alpha-smooth muscle actin, and E-cadherin. After primary antibody incubation, the blots were treated with secondary antibodies at an appropriate dilution. Visualization was achieved using the Western Lightning Plus-ECL system (PerkinElmer, Waltham, MA, USA) 29.

### Statistical analysis

Demographic characteristics and outcomes were analyzed using descriptive statistics, presenting mean and SD or median with IQR for continuous variables and counts/percentages for categorical ones. ANOVA, Kruskal-Wallis, and Chi-square tests compared variables across different CKD stages. Routine follow-ups included eGFR assessment, and changes from baseline to final follow-up were compared. Paired sample t-tests evaluated pre- and post-treatment continuous variables (eGFR, creatinine, and other blood biomarkers). IBM SPSS version 26.0 performed statistical analyses, and R software with "ggplot2" generated plots. Two-sided p-values were used, with P<0.05 indicating statistical significance.

## Results

### Astragaloside IV fingerprint in EFT

**Figure 1:**
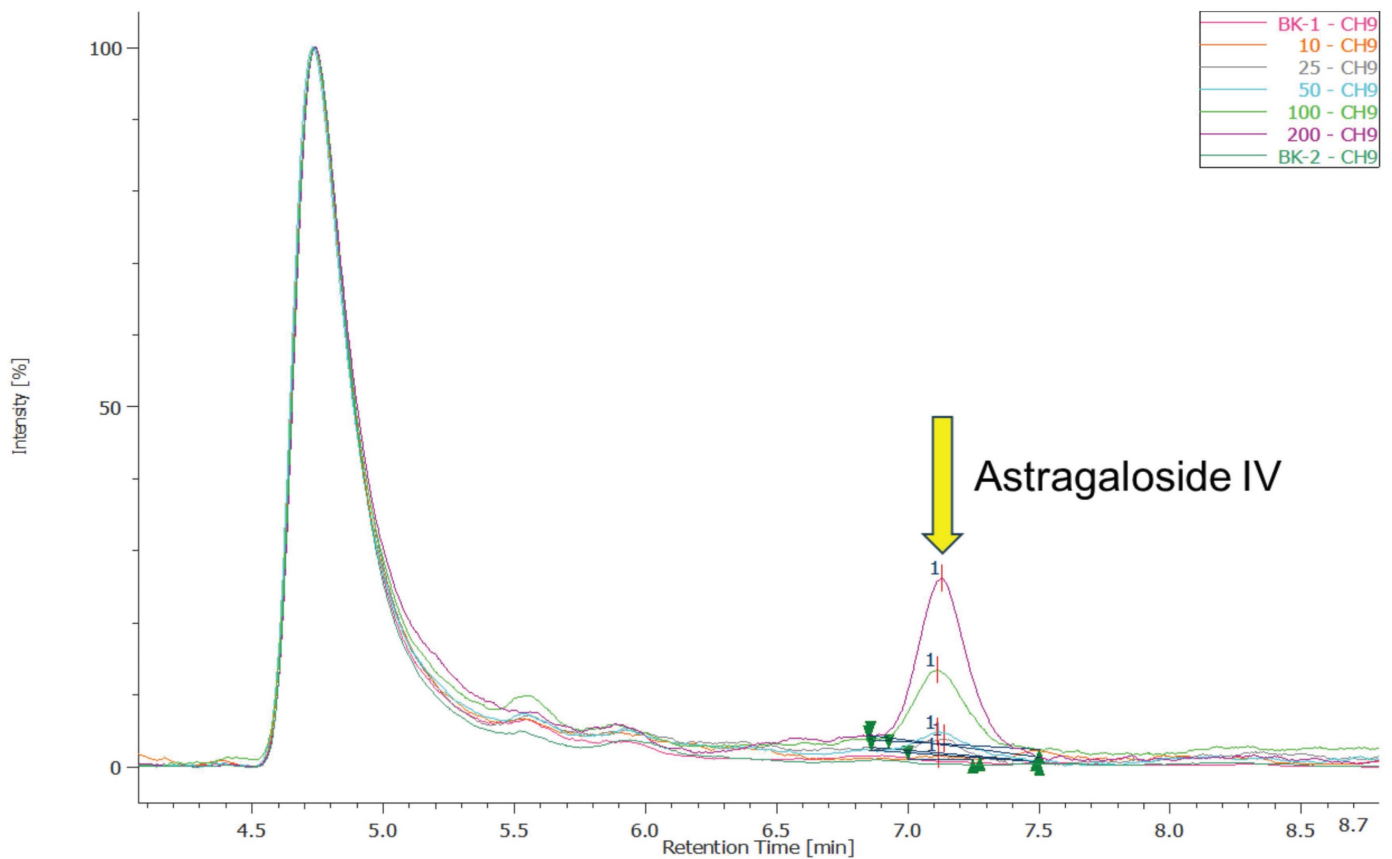
The standard curve of the Astragaloside IV. BK-1 and BK-2 represent the background solvent methanol, showing no characteristic peak at 7.1 minutes, which confirms the absence of background interference for Astragaloside IV detection. An early solvent peak appears at approximately 4.8 minutes, caused by the elution of the mobile phase solvent immediately after injection; this does not interfere with the quantification of Astragaloside IV. The standard curve for Astragaloside IV is shown on the right, with a regression equation of y=843.73x-7753.8 and a correlation coefficient R2=0.9994, demonstrating excellent linearity within this concentration range and making this method suitable for detecting samples containing Astragaloside IV.

Figure 1 shows the HPLC chromatograms of Astragaloside IV at different concentrations (10, 25, 50, 100, and 200 μg/mL) and the corresponding standard curve. In the chromatograms, a characteristic peak for Astragaloside IV is observed at a retention time of 7.1 minutes. As the concentration increases, the peak height increases proportionally, indicating a strong linear relationship between concentration and peak area. An early solvent peak appears at approximately 4.8 minutes, caused by the elution of the mobile phase solvent immediately after injection; this does not interfere with the quantification of Astragaloside IV. The standard curve for Astragaloside IV is shown on the right, with a regression equation of y=843.73x-7753.8 and a correlation coefficient R^2^=0.9994.

**Figure 2:**
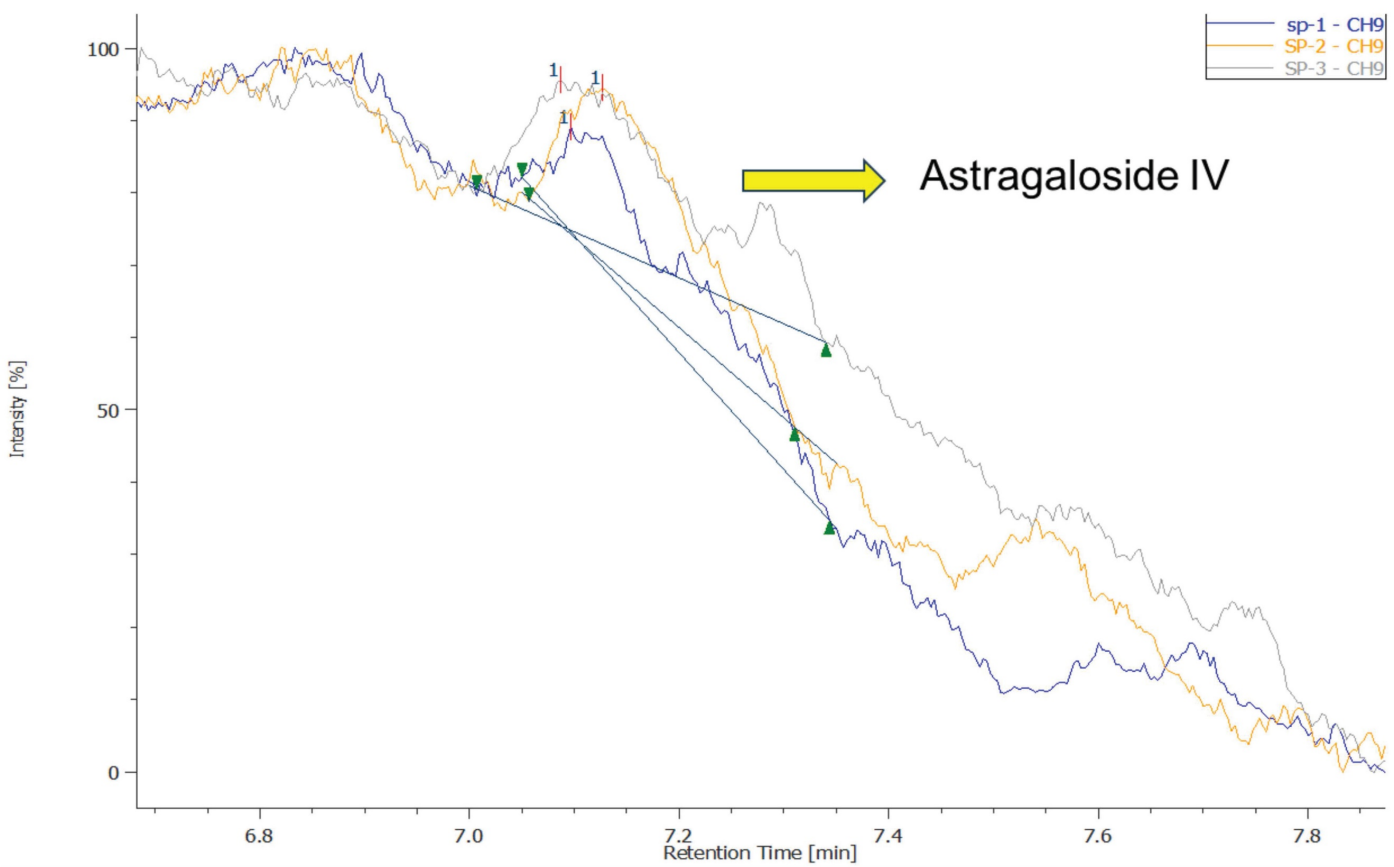
The concentration of Astragaloside IV in EFT.

Figure 2 illustrates the concentration of Astragaloside IV in EFT. Astragaloside IV, the integrated chromatogram of the triplicate samples showed a weak characteristic peak at 7.1 minutes, confirming the presence of Astragaloside IV in the EFT. The signal intensity of this characteristic peak did not fall below the minimum concentration of the calibration curve (10 μg/mL), thus allowing the use of interpolation to accurately calculate the Astragaloside IV content in the samples. Further conversion indicates that the average Astragaloside IV content per 100 mg of sample was 0.172 mg, corresponding to a weight percentage of 0.17%. This result demonstrates that although the Astragaloside IV content in the samples is relatively low, it is within the detectable range and shows a certain level of stability and consistency.

### Patient selection

Ninety-eight patients (46 women, 52 men) with stage 3-5 CKD received EFT alongside standard CKD medications for six months. Twenty-three participants were withdrawing; three experienced dizziness and withdrew before treatment, while others cited an unpleasant taste as the reason for discontinuation. The analysis included 75 patients.

### Characteristics of the patients

Table 1 presents the baseline characteristics of the analyzed 75 patients (33 females, 42 males; mean age: 68.20 years; range: 20 to 80 years). No significant differences were observed in sex (P=0.602), age (P=0.234), body weight (P=0.439), mean corpuscular volume (MCV) (P=0.385), mean corpuscular hemoglobin (MCH) (P=0.881), mean corpuscular hemoglobin concentration (MCHC) (P=0.280), and urine creatinine (P=0.148) among patients with different CKD stages. However, significant differences were noted in albumin (P=0.002) and hemoglobin (P<0.001).

Due to variance heterogeneity in urine protein-to-creatinine Ratio (UPCR) and eGFR, multiple nonparametric tests were conducted, revealing statistically significant differences in UPCR (P<0.001) and eGFR (P<0.001) across various CKD stages.

### Change in eGFR

Table 2 summarizes the findings, indicating a significant enhancement in patients' eGFR at 6 months post-treatment compared to baseline values (34.37 ± 13.58 ml/min/1.73 m^2^ vs. 42.47 ± 18.82 ml/min/1.73 m^2^, p <0.001). Both right and left kidney sizes increased from baseline to month 6 without pathological enlargement.

Stratified analysis by CKD stages, as presented in Table 2, demonstrates a noteworthy eGFR improvement in patients with CKD stage 3 (42.37 ± 6.64 ml/min/1.73 m2 vs. 51.90 ± 13.41 ml/min/1.73m^2^, p<0.001) and stage 4 (24.18 ± 4.74 ml/min/1.73 m^2^ vs. 29.28 ± 10.23 ml/min/1.73m^2^, p=0.037).

### Change in blood biomarkers

At 6 months post-treatment, creatinine levels significantly decreased in patients with CKD stage 3 (1.56 ± 0.26 vs. 1.35 ± 0.31, p<0.001) and stage 5 CKD (7.33±2.69 vs. 5.58±2.41, p=0.006), as depicted in Table 2.

Table 2 illustrates that HbA1c levels remained unchanged before and after 6 months of treatment for all patients and those stratified by CKD stages (all P-values > 0.05). Additionally, no significant differences were observed in hemoglobin levels before and after the 6-month treatment for both overall patients and those stratified by CKD stages (all P-values >0.05).

Serum potassium and sodium levels showed no significant changes among CKD patients overall before and after EFT combination treatment, as indicated in Table 2. Calcium and phosphorus levels also exhibited no significant alterations. Moreover, levels of GOT and GPT demonstrated no significant differences at each follow-up visit compared to the baseline.

### Change in kidney size

Table 3 displays ultrasonography scans depicting bilateral kidney size before and after treatment. In Table 3, the average longitudinal lengths of the left kidney and right kidney were 8.92 ± 1.15 and 8.85 ± 1.24 cm, while the average lateral lengths were 4.45 ± 0.61 and 4.48 ± 0.71, respectively. Following the 6-month treatment, both the lateral width and longitudinal length of the kidneys increased. By month 6, the mean longitudinal lengths of the left and right kidneys were 9.36 ± 1.06 and 9.26 ± 1.11 cm, respectively, and the average lateral lengths increased to 4.59 ± 0.65 and 4.71 ± 0.80 cm. These changes were statistically significant (p<0.05), except for the lateral width of the left kidney, which showed marginal significance (p=0.051).

### Safety issues

As mentioned earlier, three patients reported dizziness and withdrew from the study before receiving any treatment. Other participants who discontinued the study attributed their withdrawal to an unpleasant taste. Notably, no gastrointestinal discomfort or other adverse events related to the treatment were observed among the included patients.

### IS decreases HK-2 cell viability, but EFT enhances HK-2 cell viability

**Figure 3:**
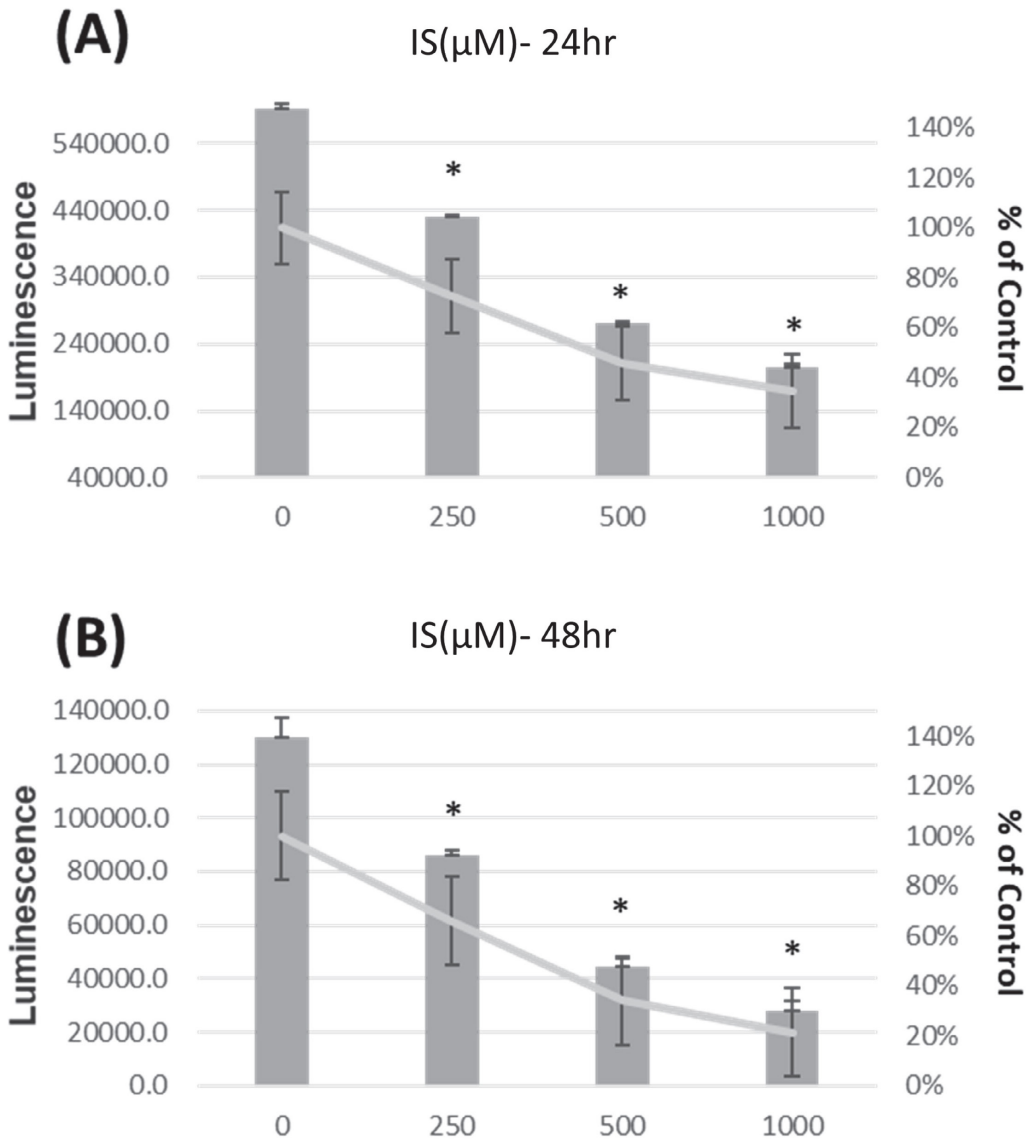
The viability of HK-2 cells is presented after treatment with IS at 0, 250, 500, and 1000 µM for 24 hours (Panel A) and 48 hours (Panel B). *p < 0.05 when compare with 0 µM of IS, n=6.

In Figure 3, the viability of HK-2 cells is presented after treatment with IS at 0, 250, 500, and 1000μM for 24 hours (Panel A) and 48 hours (Panel B), showing a dose-dependent decrease in viability.

**Figure 4:**
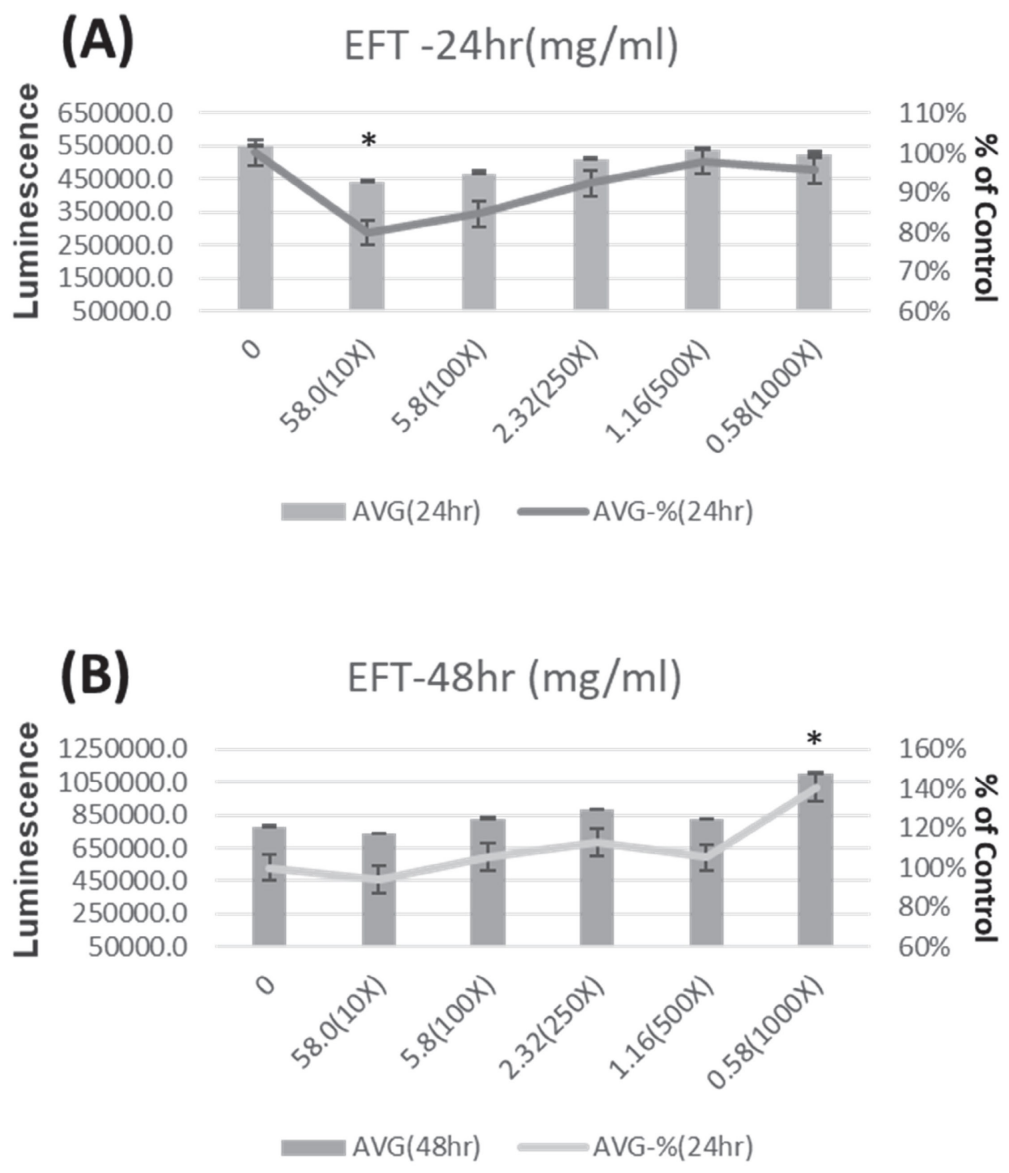
The viability of HK-2 cells after treatment with EFT at various concentrations (0mg/ml, 58.0 mg/ml (Dilute the stock solution 10 times), 5.8 mg/ml (Dilute the stock solution 100 times), 2.32 mg/ml (Dilute the stock solution 250 times), 1.16 mg/ml (Dilute the stock solution 500 times), and 58 mg/ml (Dilute the stock solution 1000 times) for 24 hours (Panel A) and 48 hours (Panel B). *p < 0.05 when compare with 0 µM of EFT, n=6.

Figure 4 shows the viability of HK-2 cells after treatment with different concentrations of EFT (0 mg/ml, 58.0 mg/ml (diluted in the original solution 10 times), 5.8 mg/ml (diluted in the original solution 100 times), 2.32 mg/ml) ml (diluted Stock solution 250 times), 1.16 mg/ml (stock solution diluted 500 times) and 58 mg/ml (stock solution diluted 1000 times)) at 24 hours (Figure 4A) and 48 hours (Figure 4B). HK-2 cells treated with different concentrations of EFT showed increased viability after 24 and 48 hours. Briefly, the viability of HK-2 cells increased at all different EFT concentrations.

### EFT enhances the clonogenicity of HK-2 cells

**Figure 5:**
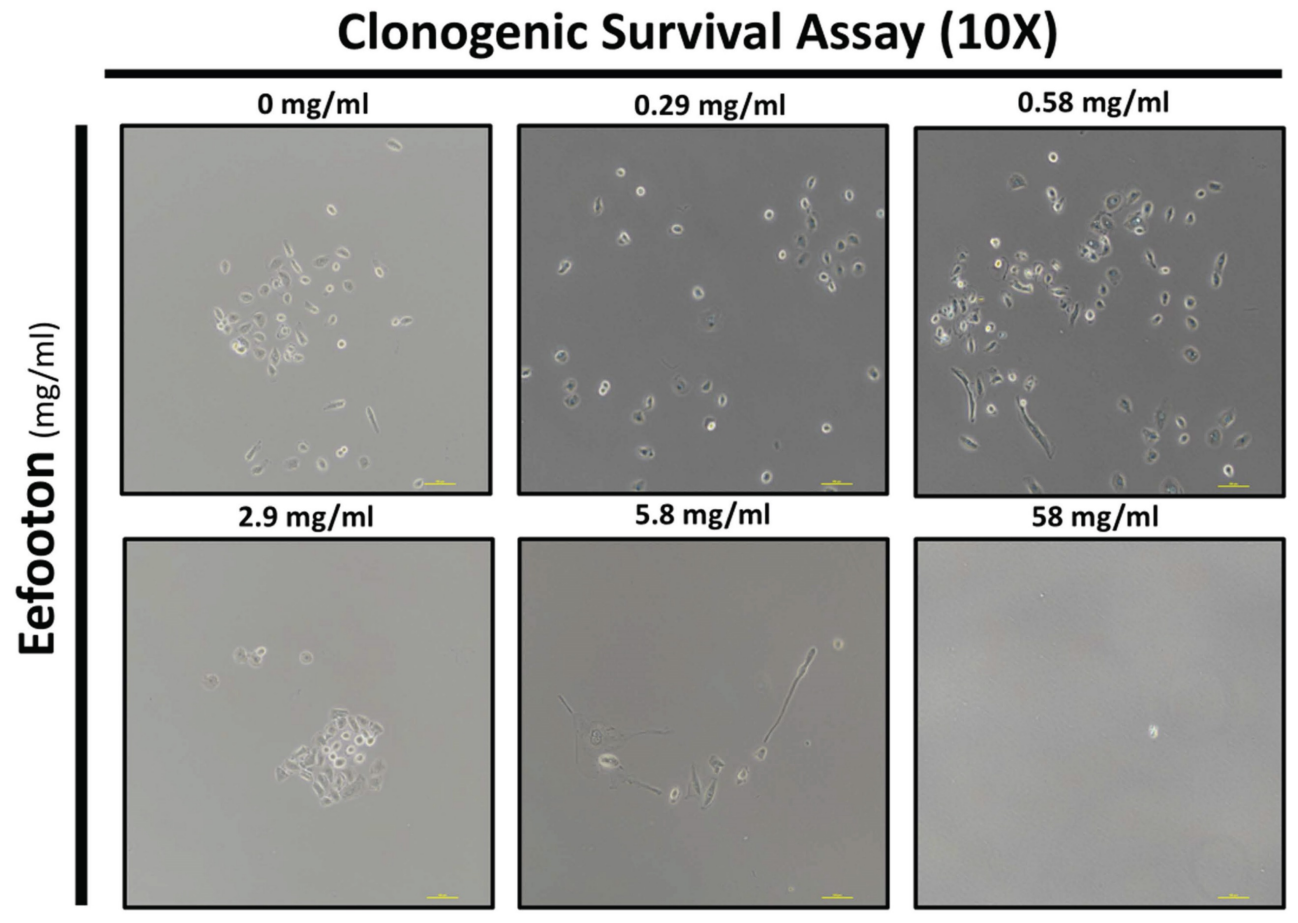
EFT enhances the clonogenicity of HK-2 cells. The light phase images illustrate the colony status mediated by EFT at various concentrations (0 mg/ml, 0.29 mg/ml, 0.58 mg/ml, 2.9 mg/ml, 5.8 mg/ml, and 58 mg/ml).

Figure 5 presents the light phase images illustrating the colony status mediated by EFT at various concentrations (0 mg/ml, 0.29 mg/ml, 0.58 mg/ml, 2.9 mg/ml, 5.8 mg/ml, and 58 mg/ml). At 0 mg/ml, no colony aggregates were observed, indicating a lack of colony formation at this concentration. Significantly, colony formation became evident at specific concentrations, particularly at 2.9 mg/ml and 0.58 mg/ml.

### EFT diminishes the expression of fibrosis-related proteins induced by IS

**Figure 6:**
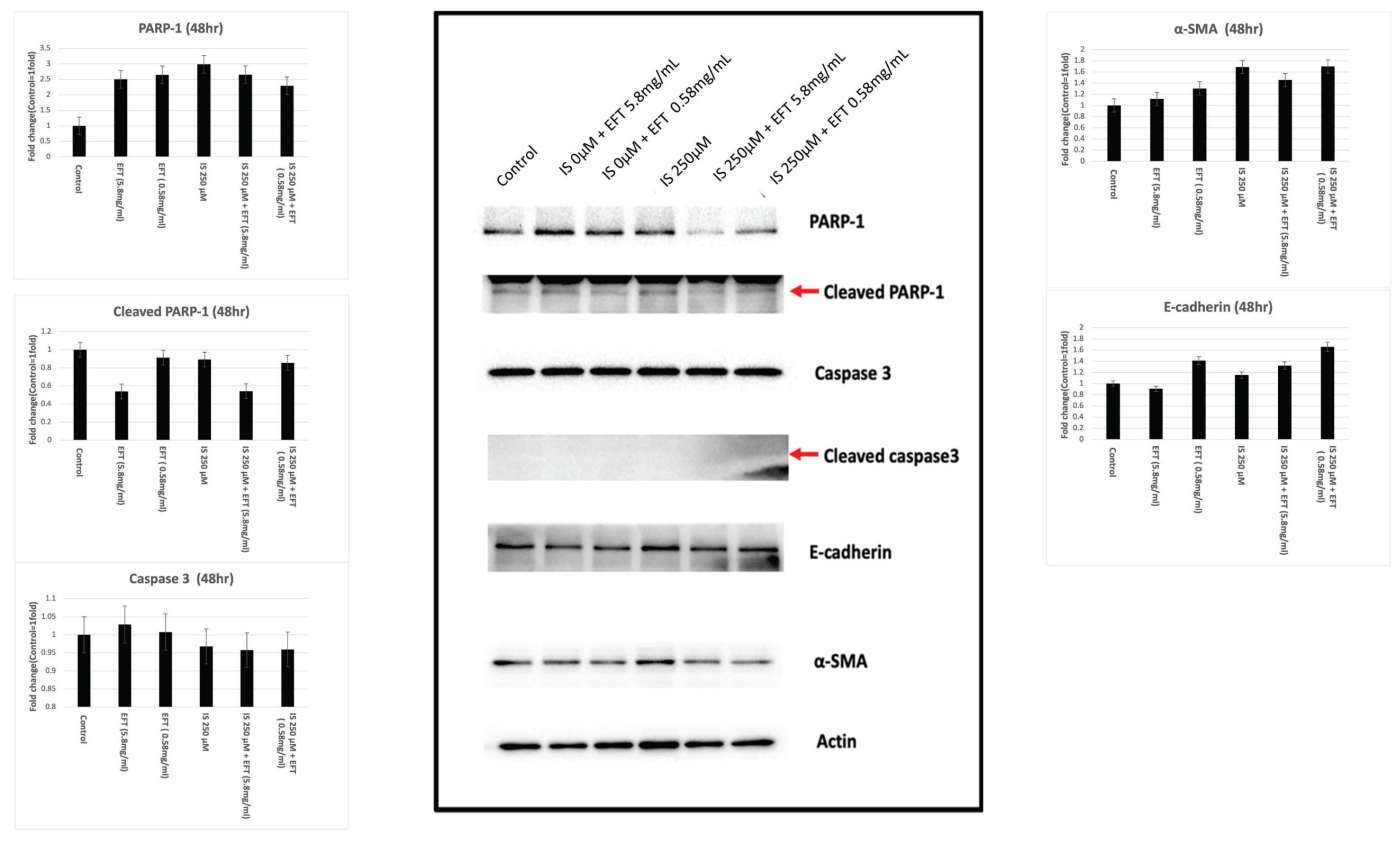
The western blot analysis of the HK-2 cells treated with IS and EFT in different concentration. Western blot analysis for the activity of proteins related to apoptosis (caspase 3, PARP-1) and fibrosis (α-smooth muscle actin (α-SMA), E-cadherin). In HK-2 cells treated with 250 µM IS, the cleaved and total PARP-1 activity was increased. Co-treatment with IS and EFT at 5.8 mg/ml and 0.58 mg/ml resulted in decreased cleaved PARP-1 activity. α-SMA activity increased at 250 µM IS, but decreased in IS and EFT co-treated HK-2 cells at 5.8 mg/ml and 0.58 mg/ml, suggesting a potential inhibitory role of EFT in fibrosis-related processes.

Figure 6 illustrates the activity of proteins related to apoptosis (caspase 3, PARP-1) and fibrosis (α-smooth muscle actin (α-SMA), E-cadherin). In HK-2 cells treated with 250 μM IS, the cleaved and total PARP-1 activity increased compared to 0 μM. However, co-treatment with IS and EFT at 5.8 mg/ml and 0.58 mg/ml resulted in decreased cleaved PARP-1 activity, suggesting potential attenuated PARP-1 activity with EFT treatment. α-SMA activity increased at 250μM IS, but decreased in IS and EFT co-treated HK-2 cells at 5.8 mg/ml and 0.58 mg/ml, indicating a potential inhibitory role of EFT in fibrosis-related processes.

Caspase 3 and E-cadherin activities were similar across different conditions, suggesting no significant alterations in apoptosis-related processes under the experimental conditions.

**Figure 7:**
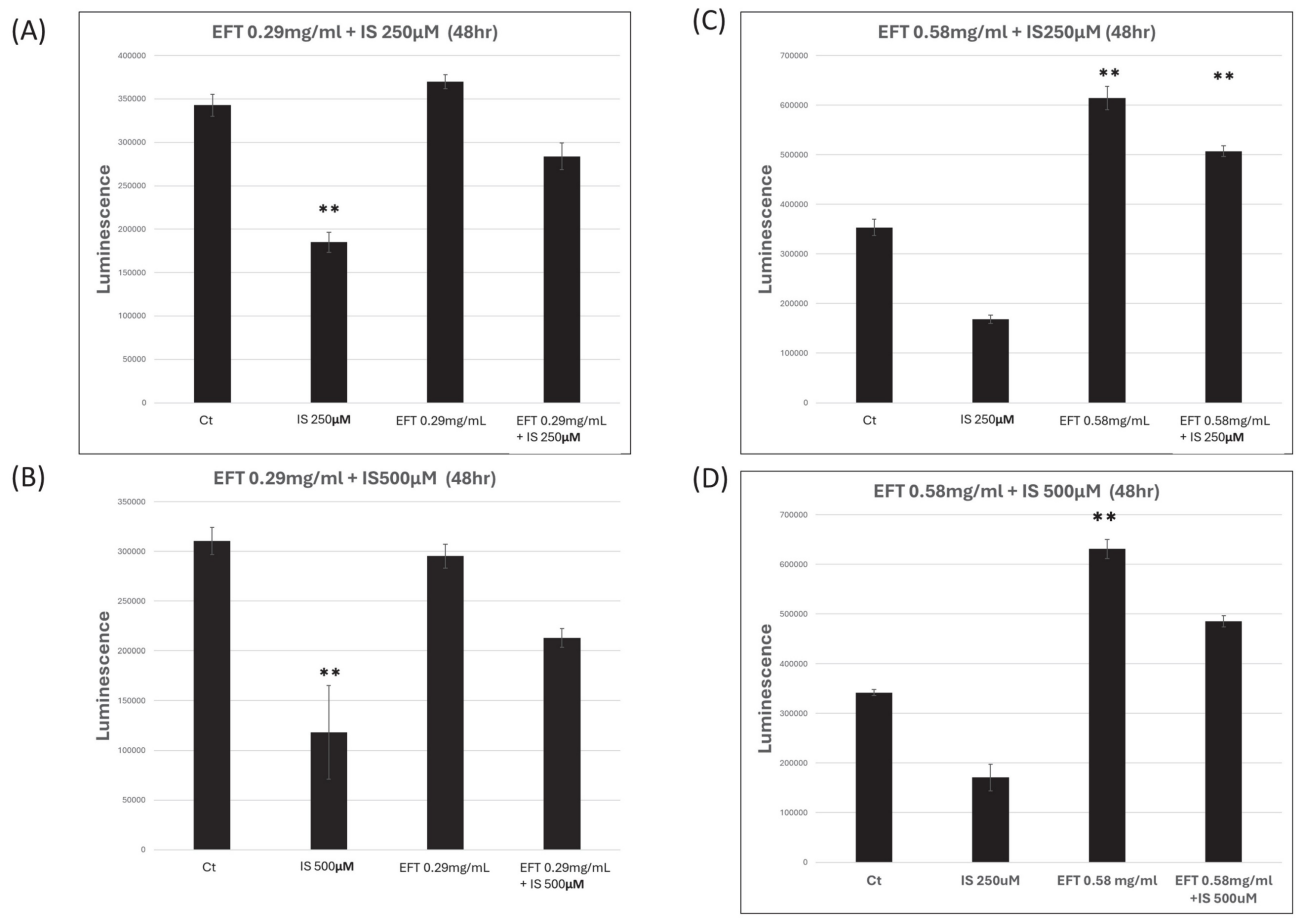
The viability of HK-2 cells after combination of EFT and IS at 48 hours. Panel A and B revealed the EFT at 0.29 mg/mL for the HK-2 cells with IS at 250 and 500 μM for 48 hours. Panel C and D revealed the EFT at 0.58 mg/mL for the HK-2 cells with IS at 250 and 500 μM for 48 hours. **: p < 0.05 when compare with 0 µM of EFT and IS, n=6.

Figure 7 illustrates the effect of EFT on the viability of the IS-treated HK-2 cells at different concentrations. Panel A and B revealed the EFT at 0.29 mg/mL for the HK-2 cells with IS at 250 and 500 μM for 48 hours. The cell viability was similar in the control group and EFT with IS group, while the viability in the IS-treated group was lower than in the control group (p<0.05). Panel C and D revealed the EFT at 0.58 mg/mL for the HK-2 cells with IS at 250 and 500μM for 48 hours. The cell viability increased in the EFT group and EFT with group in comparison with the control group (p<0.05).

## Discussion

Numerous studies have explored the potential of herbs in treating various human ailments, including CKD 30-33. This study demonstrated a significant improvement in estimated glomerular filtration rate (eGFR) in stage 3-5 CKD patients after six months of EFT supplementation alongside conventional CKD management. No adverse events were observed, and liver function markers (GOT and GPT) showed no significant changes, suggesting EFT did not adversely affect the liver. The observed improvement may be attributed to EFT's protective effects against uremic toxins, which attenuate renal tubular cell viability and promote renal fibrosis.

After 6 months of treatment, patients' kidneys enlarged significantly without signs of tumors, kidney stones, hydronephrosis, or compensatory hypertrophy 34, 35. This suggests a potential impact of combining EFT with conventional medications on nephrons or glomeruli in CKD, a noteworthy observation given the usual rapid progression of kidney atrophy in CKD. CKD in clinical practice is often associated with reduced kidney size, cortical thickness, renal fibrosis, and atrophy due to nephrosclerosis, leading to a decreased number of functional glomeruli 36. Tubulointerstitial fibrosis, a common feature in progressive CKD, further contributes to nephron atrophy and renal fibrosis 37. Typically, kidney atrophy is considered irreversible 38. While additional biopsies are necessary to unveil the underlying mechanisms, these findings offer valuable insights into potential interventions for tubulointerstitial fibrosis in CKD patients. The synergetic effect of different compounds was measured in different organs. Chu *et al.* illustrated the synergic protection of *Astragalus* and *Codonopsis pilosula* in alveolar macrophage when confronted with PM2.5 39. The combination of Astragalus and *Codonopsis pilosula* also improved the immunity and antioxidative capacity in pigs 40. The synergic reaction on immunomodulation was also documented in the combination of *Astragalus membranaceus* (AM) and *Ligustrum lucidum in macrophage* 41. However, the combination of various compounds on the renoprotection is lacking. Our study provided a combination of Astragaloside IV with other compounds in renoprotection. In advanced CKD, sustained inflammation and fibrosis progress resulted in extensive scarring and hardening of kidney tissues. This ultimately leads to end-stage renal disease, where such treatments are less effective. In the early stages of chronic kidney disease (CKD), inflammation and fibrosis in the kidneys begin subtly. Inflammatory cells release cytokines and growth factors, leading to initial tissue scarring. At the early stage of kidney disease, anti-inflammatory, antioxidant, and anti-fibrotic treatments may help preserve kidney function or even partially reverse damage 42. The progression of fibrosis further diminishes kidney function in a self-perpetuating cycle. The diverse components of EFT, known for their potent anti-inflammatory, antioxidant, and anti-fibrotic properties, help to slow down kidney function deterioration, especially in early CKD, as demonstrated in our study.

Deviations in mineral homeostasis elevate the risk of cardiovascular diseases, contributing to heightened mortality during the progression of CKD 43. Notably, in this study, a 6-month EFT treatment did not alter serum potassium, sodium, calcium, and phosphorus levels. This suggests that EFT does not influence serum mineral balance and may potentially contribute to maintaining mineral homeostasis.

In our previous study, EFT effectively improved renal, cardiac, and hepatic functions in CKD patients, showcasing its anti-inflammatory, antioxidant, and anti-fibrotic properties 4, 5. Comprising five herbal extracts, EFT's main bioactive substances are flavonoids, terpenoids, and polysaccharides, which possess various beneficial properties 44-46. The formulation leverages the synergistic effects of these components 47. For example, *Codonopsis pilosula* contains active metabolites with potent antioxidant effects, used to treat conditions like anemia, fatigue, and diabetes 4. Its polysaccharides also boost immunity in CKD patients by enhancing hematopoiesis 48. Astragaloside IV, extracted from *Astragalus membranaceus*, inhibits renal fibrosis by reducing oxidative stress and inflammation via the TGF-β/Smad pathway 49, 50. Salidroside in EFT, derived from *R. sacra*, acts as an antioxidant, reducing oxidative stress and protecting the kidneys 51. *P. quinquefolius* contains dammarane-type ginsenosides, enhancing glomerular function, preventing ECM accumulation, inhibiting apoptosis, and reducing podocyte damage for kidney protection 20. Astragaloside IV has been extensively studied in the CKD model compared to other compounds 49, 52-54. Therefore, we regarded astragaloside IV as the major compound for footprint in EFT and performed HPLC to calculate the concentration in EFT.

In our cellular study, viability decreased dose-dependently with indoxyl sulfate (IS). However, viability increased at 24 and 48 hours with EFT treatment. Colony formation exhibited a concentration-dependent effect by EFT. This concentration-specific response may have implications for understanding the biological effects of EFT. The exposure to IS increases both cleaved and total PARP-1 activity. Co-treatment with EFT and IS results in decreased cleaved PARP-1 activity, indicating a potential attenuation of PARP-1 activity by EFT. Previous research highlights the significance of PARP-1 activity in proximal renal tubular cells in kidney disease. In CKD, there is increased cytochrome C leakage and enhanced PARP-1 cleavage associated with cellular apoptosis 55. PARP-1 plays a role in DNA repair during mild damage and is inhibited by caspases in moderate DNA damage, leading to controlled cell death. Extensive DNA disruption causes over-activation of PARP-1 and cellular necrosis, but PARP inhibition attenuates this damage 56, 57. Understanding and targeting PARP-1 activity by EFT could be a therapeutic strategy for CKD, especially considering the vulnerability of proximal tubules in renal function. In addition, alpha-SMA activity increased with indoxyl sulfate (IS) exposure, indicating a potential role in fibrosis. In the presence of both IS and EFT, there was a decrease in alpha-SMA activity, implying that EFT efficiently prevents IS-induced fibrosis. The attenuation of the fibrosis process may contribute to the protective effects of EFT on renal function deterioration. However, the caspase 3 and E-cadherin activities remained similar across conditions, indicating no significant alterations in apoptosis-related processes under the experimental conditions. The current work explores the potential benefits of EFT, specifically its PARP-1 inhibition and anti-fibrosis properties, in the context of CKD.

Current CKD treatments are inadequate, leading patients to explore complementary options like TCM, including EFT 58. With EFT's diverse mechanisms in CKD treatment, there's a demand for robust clinical trials and pharmacological studies. Integrating herbal medicine with Western approaches for CKD is gaining interest 30. Further research is essential to understand molecular mechanisms, optimal dosages, and treatment durations with EFT.

### Strengths and limitations

This study represents the initial assessment of EFT's efficacy and safety in combination with conventional medications for pre-dialysis CKD patients 59. This pilot study's limited sample size affects its representativeness. Future research will use larger samples and diverse study designs, including double-blind, placebo-controlled trials. Uncertainties about the long-term impact of a 6-month EFT regimen will be addressed in subsequent studies over 1-2 years. Besides, the variation of proteinuria before and after intervention was not measured in the study. The risk of proteinuria on mortality and other major cardiovascular events was documented by the KDIGO guideline and other studies 60. Due to the small sample size, future investigations will evaluate EFT's effectiveness in different subpopulations, various time intervals, and the severity of the proteinuria. A comprehensive animal study, including renal histology examination and further exploration of molecular signaling pathways, is necessary to understand the precise benefits to the kidney. Besides, the compounds in EFT provided the anti-inflammatory effect in the basic research of the CKD model, but the head-to-head comparison was lacking. To compare the interactions, further studies should be initiated. Additionally, further research is essential to comprehend potential interactions between EFT and other medications.

## Conclusions

This study highlights the potential of EFT as an adjuvant therapy for CKD patients. Combining EFT with conventional treatment significantly improved eGFR, particularly for stage 3 CKD patients. The research also observed a reversal in kidney atrophy, a common CKD manifestation, indicating potential benefits for nephron or glomeruli health. These findings imply that EFT could potentially delay the initiation of early dialysis. The observed positive effects are likely due to EFT's ability to enhance tubular cell viability and reduce fibrosis.

## Figures and Tables

**Table 1:** Demographic and clinical characteristics of the patients with stage 3-5 CKD.

Characteristics	All (n =75)	Stage 3 (n =50)	Stage 4 (n =16)	Stage 5 (n =9)	P value
Sex					0.602
Male	42(56.0%)	26 (61.90%)	10 (23.81%)	6 (14.29%)	
Female	33(44.0%)	24 (72.73%)	6 (18.18%)	3 (9.09%)	
Age (years)	68.20±10.76	66.90±9.98	72.19±10.92	68.33±13.95	0.234
Weight (kg)	65.84±14.11	64.43±13.01	67.75±14.10	70.27±19.80	0.439
Albumin (g/dL)	4.41±0.38	4.50±0.30	4.13±0.52	4.34±0.25	**0.002**
Hemoglobin (g/dL)	12.26±2.08	12.95±2.02	11.18±1.53	10.37±1.18	**<0.001**
MCV (fL)	91.90±8.29	91.60±9.01	90.88±7.39	95.42±4.34	0.385
MCH (pg)	30.12±3.27	30.09±3.53	29.93±3.32	30.61±1.23	0.881
MCHC (g/dL)	32.72±1.25	32.79±1.24	32.86±1.42	32.10±0.90	0.280
Urine creatinine (mg/dL)	82.28±56.83	89.15±60.59	79.41±54.74	49.21±14.86	0.148
UPCR	557(50-10910)	384.50(50-10274)	2200.50(66-10910)	1018(418-9780)	**<0.001**
eGFR (mL/min/1.73m^2^)	36.70(4-58)	41.85(30.70-58)	24.55(16.10-29.80)	7.50(4-12)	**<0.001**

Abbreviations: CKD, chronic kidney disease; MCV, mean corpuscular volume; MCH, mean corpuscular hemoglobin; MCHC, mean corpuscular hemoglobin concentration; UPCR, urine protein-to-creatinine ratio; eGFR, estimated glomerular filtration rate. Chi-square test: Sex. ANOVA: Age, weight, albumin, hemoglobin, MCV, MCH, MCHC. Kruskal-Wallis test: UPCR, eGFR.

**Table 2:** Changes in blood biomarkers of the patients with stage 3-5 CKD, before and after 6-month treatment

Characteristics	Before treatment	After treatment	*P-value*
eGFR (mL/min/1.73m^2^)	34.37±13.58	42.47±18.82	<0.001
Stage 3	42.37±6.64	51.90±13.41	<0.001
Stage 4	24.18±4.74	29.28±10.23	0.037
Stage 5	8.03±2.47	13.52±11.43	0.142
BUN (mg/dL)	34.13±19.58	33.59±18.19	0.858
Creatinine (mg/dL)	2.48±2.08	2.10±1.72	<0.001
Stage 3	1.56±0.26	1.35±0.31	<0.001
Stage 4	2.63±0.62	2.49±1.39	0.631
Stage 5	7.33±2.69	5.58±2.41	0.006
Albumin (g/dL)	4.41±0.38	4.36±0.41	0.466
HbA1c (%)	6.00±1.17	5.90±1.35	0.206
Hemoglobin (g/dL)	12.26±2.08	12.06±2.27	0.135
GOT (U/L)	22.03±11.53	21.76±13.52	0.899
GPT (U/L)	17.26±9.03	16.83±9.17	0.776
WBC (10^3^/uL)	6.61±1.54	6.20±1.62	0.110
Na (mmol/L)	138.59±2.94	146.78±69.57	0.610
K (mmol/L)	4.39±0.55	4.46±0.55	0.640
Ca (mmol/L)	9.37±0.60	9.22±0.64	0.392
P (mmol/L)	3.83±0.86	3.98±0.66	0.489

Abbreviations: CKD, chronic kidney disease; BUN, blood urea nitrogen; HbA1c, glycated hemoglobin; GOT, glutamate oxaloacetate transaminase; GPT, glutamate pyruvate transaminase; WBC, white blood cell; eGFR, estimated glomerular filtration rate. Paired sample t-test.

**Table 3:** Changes in kidney size in patients with stage 3-5 CKD, before and after 6-month treatment.

	Before	After	|T|	*p-value*
Longitudinal length of left kidney (cm)	44.53±6.06	45.87±6.45	-1.99	0.051
Longitudinal length of right kidney (cm)	44.84±7.07	47.11±8.01	-3.15	0.002
Lateral width of left kidney (cm)	89.21±11.54	93.64±10.56	-3.86	<0.001
Lateral width of right kidney (cm)	88.49±12.35	92.57±11.09	-4.13	<0.001

Paired sample t-test.

## Abbreviations

CKD: Chronic kidney disease
TCM: traditional Chinese medicine
eGFR: estimated glomerular filtration rate
ESKD: end-stage kidney disease
DDIs: drug-drug interactions
MCV: mean corpuscular volume
MCH: mean corpuscular hemoglobin
MCHC: mean corpuscular hemoglobin concentration

## References

[1] Kidney Disease (2022). Improving Global Outcomes Diabetes Work G. KDIGO 2022 Clinical Practice Guideline for Diabetes Management in Chronic Kidney Disease. Kidney Int.

[2] Chen HY, Pan HC, Chen YC, Chen YC, Lin YH, Yang SH (2019). Traditional Chinese medicine use is associated with lower end-stage renal disease and mortality rates among patients with diabetic nephropathy: a population-based cohort study. BMC complementary and alternative medicine.

[3] Wang Y, Feng Y, Li M, Yang M, Shi G, Xuan Z (2022). Traditional Chinese Medicine in the Treatment of Chronic Kidney Diseases: Theories, Applications, and Mechanisms. Frontiers in pharmacology.

[4] Yao CA (2022). Case Report Eefooton adjuvant therapy for diabetic nephropathy, heart failure, and pulmonary effusion: a case report and literature review. Int J Clin Exp Med.

[5] Yao CA, Lin CH (2019). Treatment with the herbal formulation Eefooton slows the progression of chronic kidney disease: A case report. Medicine.

[6] Li JW, Vederas JC (2009). Drug discovery and natural products: end of an era or an endless frontier?. Science.

[7] Gurib-Fakim A (2006). Medicinal plants: traditions of yesterday and drugs of tomorrow. Mol Aspects Med.

[8] Lin MY, Chiu YW, Chang JS, Lin HL, Lee CT, Chiu GF (2015). Association of prescribed Chinese herbal medicine use with risk of end-stage renal disease in patients with chronic kidney disease. Kidney Int.

[9] Zhang HW, Lin ZX, Xu C, Leung C, Chan LS (2014). Astragalus (a traditional Chinese medicine) for treating chronic kidney disease. Cochrane Database Syst Rev.

[10] You H, Lu Y, Gui D, Peng A, Chen J, Gu Y (2011). Aqueous extract of Astragali Radix ameliorates proteinuria in adriamycin nephropathy rats through inhibition of oxidative stress and endothelial nitric oxide synthase. Journal of ethnopharmacology.

[11] Zhang HH, Liu J, Lv YJ, Jiang YL, Pan JX, Zhu YJ (2020). Changes in Intestinal Microbiota of Type 2 Diabetes in Mice in Response to Dietary Supplementation With Instant Tea or Matcha. Can J Diabetes.

[12] Tang JL, Xin M, Zhang LC (2022). Protective effect of Astragalus membranaceus and Astragaloside IV in sepsis-induced acute kidney injury. Aging (Albany NY).

[13] Shen Z, Cui T, Liu Y, Wu S, Han C, Li J (2023). Astragalus membranaceus and Salvia miltiorrhiza ameliorate diabetic kidney disease via the "gut-kidney axis". Phytomedicine.

[14] Zeng X, Li J, Lyu X, Chen J, Chen X, Guo S (2021). Untargeted Metabolomics Reveals Multiple Phytometabolites in the Agricultural Waste Materials and Medicinal Materials of Codonopsis pilosula. Front Plant Sci.

[15] Li Z, Zhu L, Zhang H, Yang J, Zhao J, Du D (2012). Protective effect of a polysaccharide from stem of Codonopsis pilosula against renal ischemia/reperfusion injury in rats. Carbohydrate polymers.

[16] Lin HM, Yen FL, Ng LT, Lin CC (2007). Protective effects of Ligustrum lucidum fruit extract on acute butylated hydroxytoluene-induced oxidative stress in rats. Journal of ethnopharmacology.

[17] Zhang JL, Du C, Poon CC, He MC, Wong MS, Wang NN (2023). Structural characterization and protective effect against renal fibrosis of polysaccharide from Ligustrum lucidum Ait. Journal of ethnopharmacology.

[18] Han MS, Han IH, Lee D, An JM, Kim SN, Shin MS (2016). Beneficial effects of fermented black ginseng and its ginsenoside 20(S)-Rg3 against cisplatin-induced nephrotoxicity in LLC-PK1 cells. J Ginseng Res.

[19] Hasegawa H, Sung JH, Matsumiya S, Uchiyama M (1996). Main ginseng saponin metabolites formed by intestinal bacteria. Planta Med.

[20] Jin D, Zhang Y, Zhang Y, Duan L, Zhou R, Duan Y (2021). Panax Ginseng C.A.Mey. as Medicine: The Potential Use of Panax Ginseng C. A.Mey. as a Remedy for Kidney Protection from a Pharmacological Perspective. Frontiers in pharmacology.

[21] Xu X, Lu Q, Wu J, Li Y, Sun J (2017). Impact of extended ginsenoside Rb1 on early chronic kidney disease: a randomized, placebo-controlled study. Inflammopharmacology.

[22] Evstatieva L, Todorova M, Antonova D, Staneva J (2010). Chemical composition of the essential oils of Rhodiola rosea L. of three different origins. Pharmacogn Mag.

[23] Mao Y, Li Y, Yao N (2007). Simultaneous determination of salidroside and tyrosol in extracts of Rhodiola L. by microwave assisted extraction and high-performance liquid chromatography. J Pharm Biomed Anal.

[24] Guo C, Li Y, Zhang R, Zhang Y, Zhao J, Yao J (2018). Protective Effect of Salidroside Against Diabetic Kidney Disease Through Inhibiting BIM-Mediated Apoptosis of Proximal Renal Tubular Cells in Rats. Frontiers in pharmacology.

[25] Go AS, Chertow GM, Fan D, McCulloch CE, Hsu CY (2004). Chronic kidney disease and the risks of death, cardiovascular events, and hospitalization. The New England journal of medicine.

[26] Gounden V, Bhatt H, Jialal I (2023). Renal Function Tests. 2023 Jul 17. In: StatPearls [Internet]. Treasure Island (FL): StatPearls Publishing; 2023 Jan-.

[27] Geng YB, Xu C, Wang Y, Zhang LW (2020). Long non-coding RNA SNHG11 promotes cell proliferation, invasion and migration in glioma by targeting miR-154-5p. Eur Rev Med Pharmacol Sci.

[28] Franken NA, Rodermond HM, Stap J, Haveman J, van Bree C (2006). Clonogenic assay of cells in vitro. Nat Protoc.

[29] Zhai Z, Yang F, Xu W, Han J, Luo G, Li Y (2022). Attenuation of Rheumatoid Arthritis Through the Inhibition of Tumor Necrosis Factor-Induced Caspase 3/Gasdermin E-Mediated Pyroptosis. Arthritis Rheumatol.

[30] Borkar P, Yadav V, Tiwari RR, Samarth RM (2022). A systematic review of potential candidates of herbal medicine in treatment of chronic kidney disease. Phytomedicine Plus.

[31] Zhong Y, Menon MC, Deng Y, Chen Y, He JC (2015). Recent Advances in Traditional Chinese Medicine for Kidney Disease. American journal of kidney diseases: the official journal of the National Kidney Foundation.

[32] Yao T, Su W, Han S, Lu Y, Xu Y, Chen M (2022). Recent Advances in Traditional Chinese Medicine for Treatment of Podocyte Injury. Frontiers in pharmacology.

[33] Kong LY, Tan RX (2015). Artemisinin, a miracle of traditional Chinese medicine. Nat Prod Rep.

[34] Chuang TF, Hung HC, Li SF, Lee MW, Pai JY, Hung CT (2020). Risk of chronic kidney disease in patients with kidney stones-a nationwide cohort study. BMC nephrology.

[35] Pandey RK, Arya TV, Kumar A, Yadav A (2017). Effects of 6 months yoga program on renal functions and quality of life in patients suffering from chronic kidney disease. International journal of yoga.

[36] Denic A, Glassock RJ, Rule AD (2016). Structural and Functional Changes With the Aging Kidney. Advances in chronic kidney disease.

[37] Hewitson TD, Holt SG, Smith ER (2017). Progression of Tubulointerstitial Fibrosis and the Chronic Kidney Disease Phenotype - Role of Risk Factors and Epigenetics. Frontiers in pharmacology.

[38] Wang K, Liu Q, Tang M, Qi G, Qiu C, Huang Y (2023). Chronic kidney disease-induced muscle atrophy: Molecular mechanisms and promising therapies. Biochemical pharmacology.

[39] Chu X, Liu XJ, Qiu JM, Zeng XL, Bao HR, Shu J (2016). Effects of Astragalus and Codonopsis pilosula polysaccharides on alveolar macrophage phagocytosis and inflammation in chronic obstructive pulmonary disease mice exposed to PM2.5. Environmental toxicology and pharmacology.

[40] Hua Y, Wei Y, Yuan Z, Niu C, Jiang C, Guo R (2024). The impact of Codonopsis Pilosulae and Astragalus Membranaceus extract on growth performance, immunity function, antioxidant capacity and intestinal development of weaned piglets. Frontiers in Veterinary Science.

[41] Lau BHS, Ong P, Tosk J (1989). Macrophage chemiluminescence modulated by Chinese medicinal herbs Astragalus membranaceus and Ligustrum lucidum. Phytotherapy Research.

[42] Ruiz-Ortega M, Lamas S, Ortiz A (2022). Antifibrotic Agents for the Management of CKD: A Review. Am J Kidney Dis.

[43] Abe M, Okada K, Soma M (2013). Mineral metabolic abnormalities and mortality in dialysis patients. Nutrients.

[44] Ko PH, Huang CW, Chang HH, Chuang EY, Tsai MH, Lai LC (2019). Identifying the functions and biomarkers of Codonopsis pilosula and Astragalus membranaceus aqueous extracts in hepatic cells. Chinese medicine.

[45] Liu J, Hu X, Yang Q, Yu Z, Zhao Z, Yi T (2010). Comparison of the immunoregulatory function of different constituents in radix astragali and radix hedysari. Journal of biomedicine & biotechnology.

[46] Zhou Y (2022). A review of the antibacterial activity and mechanisms of plant polysaccharides. Trends in Food Science & Technology.

[47] Zhou X, Seto SW, Chang D, Kiat H, Razmovski-Naumovski V, Chan K (2016). Synergistic Effects of Chinese Herbal Medicine: A Comprehensive Review of Methodology and Current Research. Frontiers in pharmacology.

[48] Fu YP, Li LX, Zhang BZ, Paulsen BS, Yin ZQ, Huang C (2018). Characterization and prebiotic activity in vitro of inulin-type fructan from Codonopsis pilosula roots. Carbohydrate polymers.

[49] Ji C, Luo Y, Zou C, Huang L, Tian R, Lu Z (2018). Effect of astragaloside IV on indoxyl sulfate-induced kidney injury in mice via attenuation of oxidative stress. BMC Pharmacol Toxicol.

[50] Zhang J, Wu C, Gao L, Du G, Qin X (2020). Astragaloside IV derived from Astragalus membranaceus: A research review on the pharmacological effects. Advances in pharmacology (San Diego, Calif).

[51] Zhong Z, Han J, Zhang J, Xiao Q, Hu J, Chen L (2018). Pharmacological activities, mechanisms of action, and safety of salidroside in the central nervous system. Drug design, development and therapy.

[52] Li D, Liu Y, Zhan Q, Zeng Y, Peng Z, He Q (2023). Astragaloside IV Blunts Epithelial-Mesenchymal Transition and G2/M Arrest to Alleviate Renal Fibrosis via Regulating ALDH2-Mediated Autophagy. Cells.

[53] Mo Y, Hu D, Yu W, Ji C, Li Y, Liu X (2023). Astragaloside IV attenuates indoxyl sulfate-induced injury of renal tubular epithelial cells by inhibiting the aryl hydrocarbon receptor pathway. Journal of ethnopharmacology.

[54] Su Y, Xu J, Chen S, Feng J, Li J, Lei Z (2022). Astragaloside IV protects against ischemia/reperfusion (I/R)-induced kidney injury based on the Keap1-Nrf2/ARE signaling pathway. Translational andrology and urology.

[55] Bigelman E, Cohen L, Aharon-Hananel G, Levy R, Rozenbaum Z, Saada A (2018). Pathological presentation of cardiac mitochondria in a rat model for chronic kidney disease. PloS one.

[56] Devalaraja-Narashimha K, Singaravelu K, Padanilam BJ (2005). Poly(ADP-ribose) polymerase-mediated cell injury in acute renal failure. Pharmacol Res.

[57] Gupta S, Hanna PE, Ouyang T, Yamada KS, Sawtell R, Wang Q (2023). Kidney function in patients with ovarian cancer treated with poly (ADP-ribose) polymerase (PARP) inhibitors. J Natl Cancer Inst.

[58] Li Q, Xing C, Yuan Y (2021). Mitochondrial Targeting of Herbal Medicine in Chronic Kidney Disease. Frontiers in pharmacology.

[59] Murakami K, Takemura T, Hino S, Yoshioka K (1997). Urinary transforming growth factor-beta in patients with glomerular diseases. Pediatric nephrology (Berlin, Germany).

[60] Inker LA, Astor BC, Fox CH, Isakova T, Lash JP, Peralta CA (2014). KDOQI US commentary on the 2012 KDIGO clinical practice guideline for the evaluation and management of CKD. Am J Kidney Dis.

